# Current Status of Endoplasmic Reticulum Stress in Type II Diabetes

**DOI:** 10.3390/molecules26144362

**Published:** 2021-07-19

**Authors:** Sagir Mustapha, Mustapha Mohammed, Ahmad Khusairi Azemi, Abubakar Ibrahim Jatau, Aishatu Shehu, Lukman Mustapha, Ibrahim Muazzamu Aliyu, Rabi’u Nuhu Danraka, Abdulbasit Amin, Auwal Adam Bala, Wan Amir Nizam Wan Ahmad, Aida Hanum Ghulam Rasool, Mohd Rais Mustafa, Siti Safiah Mokhtar

**Affiliations:** 1 Department of Pharmacology, School of Medical Sciences, Universiti Sains Malaysia, Kota Bharu 16150, Kelantan, Malaysia; maimunat001@gmail.com (S.M.); madkucai89@gmail.com (A.K.A.); aidakb@usm.my (A.H.G.R.); 2 Department of Pharmacology and Therapeutics, Ahmadu Bello University, Zaria 810107, Kaduna, Nigeria; pharmaishatu@gmail.com (A.S.); ialiyu71@gmail.com (I.M.A.); danrakarabiu@gmail.com (R.N.D.); 3 School of Pharmaceutical Sciences, Universiti Sains Malaysia, Penang 11800, Pulau Pinang, Malaysia; mohammedmmrx@gmail.com; 4 Department of Clinical Pharmacy and Pharmacy Practice, Ahmadu Bello University, Zaria 810107, Kaduna, Nigeria; 5 School of Pharmacy and Pharmacology, University of Tasmania, Hobart, TAS 7005, Australia; ibrahim.jatauabubakar@utas.edu.au; 6 Department of Pharmaceutical and Medicinal Chemistry, Kaduna State University, Kaduna 800241, Kaduna, Nigeria; mustaphalukman26@gmail.com; 7 Department of Physiology, Faculty of Basic Medical Sciences, University of Ilorin, Ilorin 240103, Kwara, Nigeria; amin.a@unilorin.edu.ng; 8 Membrane Traffic Group, Instituto Gulbenkian de Ciencia, 2784-156 Lisbon, Portugal; 9 Department of Pharmacology, College of Medicine and Health Sciences, Federal University Dutse, Dutse 720281, Jigawa, Nigeria; auwalubala30@gmail.com; 10 Department of Pharmacology and Therapeutics, Faculty of Pharmaceutical Sciences, Bayero University Kano, Kano 700241, Kano, Nigeria; 11 Biomedicine Programme, School of Health Sciences, Universiti Sains Malaysia, Kota Bharu 16150, Kelantan, Malaysia; wanamir@usm.my; 12 Department of Pharmacology, Faculty of Medicine, University of Malaya, Kuala Lumpur 50603, Malaysia; rais@um.edu.my

**Keywords:** endoplasmic reticulum, endoplasmic reticulum stress, apoptosis, homeostasis, unfolded protein response, type II diabetes

## Abstract

The endoplasmic reticulum (ER) plays a multifunctional role in lipid biosynthesis, calcium storage, protein folding, and processing. Thus, maintaining ER homeostasis is essential for cellular functions. Several pathophysiological conditions and pharmacological agents are known to disrupt ER homeostasis, thereby, causing ER stress. The cells react to ER stress by initiating an adaptive signaling process called the unfolded protein response (UPR). However, the ER initiates death signaling pathways when ER stress persists. ER stress is linked to several diseases, such as cancer, obesity, and diabetes. Thus, its regulation can provide possible therapeutic targets for these. Current evidence suggests that chronic hyperglycemia and hyperlipidemia linked to type II diabetes disrupt ER homeostasis, thereby, resulting in irreversible UPR activation and cell death. Despite progress in understanding the pathophysiology of the UPR and ER stress, to date, the mechanisms of ER stress in relation to type II diabetes remain unclear. This review provides up-to-date information regarding the UPR, ER stress mechanisms, insulin dysfunction, oxidative stress, and the therapeutic potential of targeting specific ER stress pathways.

## 1. Introduction

Diabetes mellitus, commonly known as diabetes, is one of the most complex diseases of humankind. It is a group of metabolic disorders characterized by chronic hyperglycemia due to defects in insulin secretion, insulin action, or both. The chronic hyperglycemia of diabetes is associated with the long-term damage, dysfunction, and failure of various organs, especially the eyes, kidneys, nerves, heart, and blood vessels [1]. In 2017, the incidence of adult diabetes was approximately 451 million cases, a figure projected to increase to 693 million in 2045 [2]. The prevalence of diabetes is higher in developed countries, and the incidence is significantly rising in developing nations, such as China and India [3].

There are two major types of diabetes: type I and type II diabetes. Type I diabetes, which accounts for approximately 10% of all diabetes, is a metabolic perturbation in which the immune system targets the pancreatic beta cells responsible for insulin synthesis [4]. Due to these effects, the body cannot produce enough insulin or is unable to produce any at all. This condition has been attributed to several factors, including environmental and genetic factors. In contrast, type II diabetes, also known as maturity-onset diabetes, is associated with insulin resistance and accounts for approximately 90% of all diabetes [5,6]. This condition occurs when the body cells cannot effectively utilize insulin, resulting in hyperglycemia and insulin overproduction [7]. 

Consequently, type II diabetes is becoming common in children due to the rising prevalence of obesity. Obesity is associated with metabolic dysfunction and is on the rise globally. People suffering from obesity tend to develop conditions, such as cardiovascular disease, hypertension, insulin dysfunction, and type II diabetes. Current evidence suggests that acute and chronic hyperglycemia and hyperlipidemia related to type II diabetes disrupt endoplasmic reticulum (ER) homeostasis resulting in irreversible unfolded protein response (UPR) activation and cell death.

The ER is an organelle responsible for the production, trafficking, processing, and secretion of protein; storage of calcium (Ca^2+^); and lipid production [8]. The ER balance ensures the survival, differentiation, development, and proliferation of cells [9]. As recorded in patients with obesity and diabetes, a disruption of this balance results in changes in the metabolism and ER stress [9,10,11,12]. Despite much progress being achieved in understanding the pathophysiology of UPR and ER stress, to date, the mechanisms of ER stress in relation to type II diabetes remain unclear.

Type II diabetes-mediated cellular dysfunction might start from a cell, eventually affecting the tissues, organs, and the whole system due to ER homeostasis perturbations [13]. These perturbations create a condition known as ER stress in most cells that is due to the presence of misfolded proteins in the ER lumen [9,12,14]. The activation of ER stress creates a coping mechanism called the ER stress response or UPR. The role of the UPR is to activate the expression of genes that prevent the overload of misfolded proteins and to restore ER homeostasis. 

If the UPR fails to restore ER balance, pro-apoptotic and pro-inflammatory downstream signaling pathways become activated [15]. However, the exact mechanisms of ER stress in relation to type II diabetes are not fully understood. UPR activation is attributable to the insulin dysfunction associated with type II diabetes [8]. This review provides up-to-date information regarding the ER, ER stress mechanisms, insulin dysfunction, oxidative stress, and the therapeutic potential of targeting ER stress in type II diabetes.

## 2. History of the Study of the Endoplasmic Reticulum

The endoplasmic reticulum (ER) was first observed in fibroblast-like cells by electron microscopy in 1945 [16] and was named the ER by Porter in 1954, a name still used to date [17]. It is one of the most organized eukaryotic organelles. It is a network of membranous tube-shaped structures and two-dimensional discs extending to the cytoplasmic area [18]. The ER lumen allows for the movement of molecules inside and outside the cytosol. In 1956, the ER was classified into two compartments: the rough ER (RER) and smooth ER (SER), based on the presence or absence of cytoplasmic ribosomes, respectively [19]. 

The RER performs protein secretion and biosynthesis, whereas the SER functions as a point of contact with other cellular organelles and a site for vesicle fusion, steroid secretion, lipid detoxification, and Ca^2+^ storage [20]. Shibata et al. in 2006 [21], proposed new frontiers that organize the ER into membrane structures. Based on this, ERs can be categorized into a nuclear envelope, sheet-like cisternae, and a polygonal array of tubules connected by three-way junctions [21]. These structures differ in terms of the presence of a membrane curvature comprising two different morphological areas: sheets and tubules.

**Figure 1:**
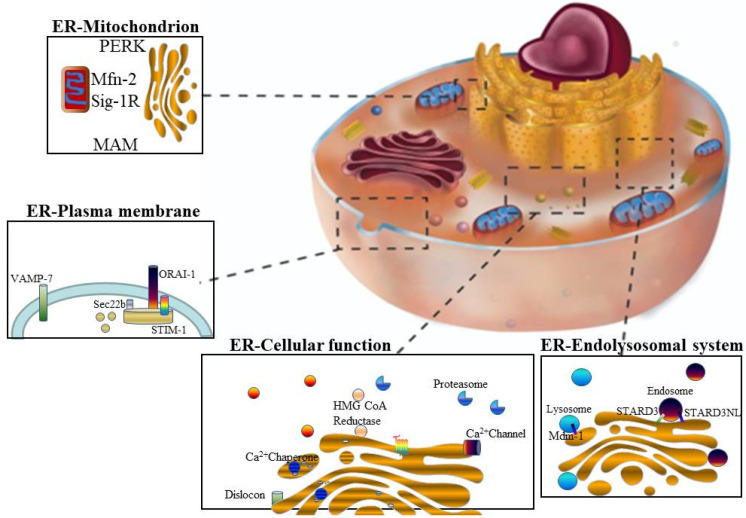
Interaction points between parts of the cell and the endoplasmic reticulum (ER). Endosomes (STARD3 and STARD3NL), lysosomes (Mdm1), mitochondria (Mfn-2, sigma-1 receptor (Sig-1R), and PERK), and PM (ORAI1, STIM1, Sec22b, and VAMP7) all generate several membrane contact sites with the ER, which has diverse physiological implications. The ER is involved in secretory, transmembrane protein folding; quality control; protein and lipid trafficking; the lipid metabolism; Ca^2+^ homeostasis; and metabolic processes. mitochondria-associated ER membrane (MAM) plays an essential role in maintaining cellular homeostasis, cell fate, signaling pathways, hormonal stimuli, and the cellular metabolism. unfolded protein response (UPR (GRP78, IRE1, and PERK)), mitofusin-2, and Sig-1R ensure the integrity of MAM and regulate ER–mitochondria communications. The ER-associated PM functions in maintaining the cellular Ca^2+^ levels via ER Ca^2+^-ATPase and plasma membrane Ca^2+^-ATPase to regulate the dynamic signaling machinery of Ca^2+^. The ER–PM association is required for muscle cell contraction and can cause STIM1 to activate ORAI1 due to ER-Ca^2+^ depletion. The vesicle-trafficking protein Sec22b, which is a non-fusogenic ER SNARE protein that can interact with the PM-SNARE Syntaxin 1 at the PM can contribute to ER–PM tethering and PM expansion in neurons. ER-associated endosomes are generated via STARD3NL, and STARD3 are the late proteins found on the endosome for MCS. STARD3NL and STARD3 are proteins that can define cholesterol-containing patches on endosomes and initiate ER–endosome contact prior to the cholesterol transfer mediated by other proteins. The lysosome MCS is Mdm-1, which helps in interoganelle tethering. Abbreviations: Vesicle-associated membrane protein 7 (VAMP7), Stromal interaction molecule 1 (STIM1), Vesicle-trafficking protein (Sec22b), Calcium release-activated calcium channel protein 1 (ORAI1), Mitofusin-2 (Mfn-2), Sigma-1 receptor (Sig-1R), Morphology 1 (Mdm1), Endosomal sterol-binding protein (STARD3), and STARD3 N-terminal-like protein (STARD3NL).

The ER interacts with many organelles within the cytoplasm, such as the mitochondria, plasma membrane (PM), endosomes, Golgi apparatus, peroxisomes, and lipid droplets, as depicted in Figure 1 [22]. The physical interaction between the ER and mitochondria is known as the mitochondria-associated ER membrane (MAM), an association that plays a crucial role in maintaining Ca^2+^ stability [23]. Mitochondria play a pivotal role in several metabolic disorders, especially type II diabetes [24]. They are the most significant source of reactive oxygen species (ROS) and are involved in cellular homeostasis, the metabolism of Ca^2+^, apoptosis, autophagy, and the production of adenosine triphosphate (ATP) [25]. 

Low levels of ROS are involved in signal transduction, whereas high levels of ROS are involved in cell damage; thus, the overproduction of ROS leads to mitochondrial disorder and a drop in the ATP output [26]. Type II diabetes-related mitochondrial disorders, insulin dysfunction, and hyperglycemia have been observed in various tissues, such as the lungs, liver, skeletal muscle, and heart [26]. Both the mitochondria and ER in MAM as ROS sources are implicated in diabetes [27,28]. 

The major consequence of type II diabetes is elevated glucose concentrations in the blood. Mitochondria use glucose as a source of energy/ATP through the electron transport chain, forming ROS as the byproduct. High ROS production due to hyperglycemia causes saturation of the antioxidant mechanisms, resulting in increased oxidative stress in the ER. The oxidative state between the ER and mitochondria results in both influencing each other in a vicious cycle through MAMs.

Autophagy is another physiological mechanism that plays a vital role in ER stress and mitochondrial function [29]. It is a physiological process that involves the self-digestion of cellular organelles or proteins mediated by stress; this process occurs in the pathogenesis of type II diabetes. Many studies have supported the cytoprotective action of autophagy in maintaining cellular balance [30]. 

ER stress activates the autophagy process due to the accumulation of misfolded proteins in the ER lumen in an attempt to restore the ER balance. The ER balance can also be restored when excess ROS are reduced through autophagy to attenuate ER stress. Furthermore, mitochondrial ROS is known as the key autophagy modulator [31]. Mitochondria exhibit a specific autophagy process called mitophagy, which is responsible for identifying and eliminating dysfunctional mitochondria that contribute to mitochondrial ROS generation in a cell [24].

The PM and ER interact via the calcium release-activated calcium channel protein 1 and stromal interaction molecule 1 (STIM1), respectively [32]. However, this interaction is balanced through vesicle-associated membrane protein 7 (VAMP7) and vesicle-trafficking protein (Sec22b) [33]. The ER and endosomes are also in contact, especially during misfolded protein accumulation [34].

The complication of type II diabetes is attributable to the disparity between the apoptosis and autophagy processes. To achieve a normal physiological function, which is required in diabetes management, the multiple molecular functions of ER with other cellular organelles must be understood. Furthermore, the next level of complexity needs to be addressed by discussing how and when the cellular organelles are formed, their organization within the cell, and their implications for diabetes. Our understanding of ER has advanced into identifying it as a cardinal organelle in endocrinology, which serves as a foundation in metabolic function. This indicates that the level of glucose in the body exhibits a profound effect on ER stability.

## 3. Discovery and Investigation of the ER Stress Mechanism

It was revealed in 1977 that sugar degradation in Rous sarcoma virus-transformed fibroblasts triggers an array of genes that result in “glucose-regulated proteins” (GRPs) [35]. In 1983, binding immunoglobulin protein (BiP) (a resident protein in ER that assists in the proper folding of proteins) was discovered to bind to immunoglobulin heavy chains in pre-B lymphocytes prior to the expression of immunoglobulin light chains [36]. Immunoglobulins or antibodies are classified into two immunoglobulins: heavy and light chains, which are connected via a disulfide bond. 

The heavy chain is classified into five immunoglobulins (IgG, IgM, IgD, IgA, and IgE), whereas the light chain is classified into two immunoglobulins (lambda (λ) and kappa (κ)). In the mid-1980s, the two proteins (GRP and BiP) were discovered to be identical, restricted to the ER lumen [37]. In the late 1980s, certain elements were discovered to prevent the stimulation of GRP gene encoding for two types of proteins: DNA damage-inducible and growth arrest proteins [38]. Furthermore, GRP gene activation was linked with the binding of misfolded and unfolded proteins to BiP/GRP [39]. The evolution of ER stress led to the discovery of its role and contributions in type II diabetes.

Yeast genetics research suggested that inositol-requiring 1 (IRE1p)-mediated splicing of HAC1mRNA generates a viable signaling pathway for UPR gene coding [40]. Tirasophon et al. in 1998 [41] showed the presence of erN1 and erN2 in mammalian yeast homologs and referred to them as IRE1α and IRE1β, respectively. Yoshida et al. in 2001 [42], demonstrated that Ire1α stimulates X-box-binding protein 1 (XBP1), resulting in downstream signaling, which leads to the generation of a functional transcription factor. This factor was identified as X-box-binding protein 1 splicing (XBP1s), which is responsible for gene up-regulation in the nucleus [43]. 

Metazoans have been used to identify two more ER stress arm sensors: activating transcription factor 6 (ATF6) and double-stranded RNA protein kinase-like ER kinase (PERK) [44,45]. The discovery of these three arm sensors (IRE1, PERK, and ATF6) serves as a golden standard in the UPR pathway mechanism. Such groundbreaking studies from the 1970s to 1990s provided foundational proof that cells have a fully integrated response mechanism triggered within the ER during stress. In fact, the upregulation of GRP78 and GRP94 has been extensively identified as proof for the ER response or UPR, which serves as a basis for the current studies on type II diabetes [46,47].

IRE1 is one of the three-arm sensors of UPR available in two kinases: IRE1α and IRE1β. IRE1α triggers many pathways via a kinase and an endonuclease in response to ER stress. The inactivation and activation of IRE1α need to be tightly controlled in the cell because a sustained activation of IRE1α leads to apoptosis. When ER stress is sensed, the UPR pathway is activated via oligomerization or dimerization of IRE1α, which eventually leads to trans-autophosphorylation [48], resulting in allosteric modifications in its configuration. 

IRE1α cleaves to specific mRNA introns by aiming at XBP1 and removes the introns of 26 nucleotides [49] to generate a stable transcription element, which is XBP1s [50]. XBP1s induces many factors for cell survival (endoplasmic reticulum-associated degradation (ERAD), ER or Golgi biogenesis, folding and secretion, translocation, and inflammation). Furthermore, when ER stress is sustained, IRE1α causes apoptosis in different ways by triggering various molecules responsible for apoptosis, especially tumor necrosis factor-alpha (TNF) receptor-associated factor 2 (TRAF2), and apoptosis signaling kinase 1 (Ask1).

PERK exists in a homodimer form under stable conditions; however, under stressful conditions, it transforms into a tetrameric structure, which results in trans-autophosphorylation of the PERK domain at the C-terminal [51]. PERK phosphorylates eIF2α at Ser51, which leads to a reduction in protein production and increases in the translation of particular sets of mRNAs, such as activation transcription factor 4 (ATF4) [52]. 

ATF4 is transcripted for the genes required to restore the cell balance, such as autophagy, molecular chaperones in ER, the lipid metabolism, the proteins necessary for optimal metabolism, redox balance, and the antioxidant response. However, when it fails to achieve the cellular balance due to sustained ER stress, ATF4 is transcripted for genes, such as enhancer-binding protein homologous protein (CHOP) and tribbles homolog 3. Both these proteins are responsible for pro-apoptotic actions [53].

ATF6 comprises a DNA transcription activation area and a basic leucine zipper (bZIP). ATF6p90 is activated due to an increase in the accumulation of misfolded and unfolded proteins in the ER lumen [43]. This leads to detachment of BiP/GRP 78 from ATF6p90, after which ATF6p90 moves to the Golgi apparatus for more processing at site 1 and site 2 of serine and metalloprotease, respectively [54]. The nucleus receives ATF6p50, which contains bZIP, from the Golgi apparatus and affects many ER stress genes to decrease ER stress. The actions of ATF6p50 and XBP1s are in the same direction. They intersect in their downstream signaling cascade to regulate the gene transcription for protein secretion, ERAD, protein folding, ER, and Golgi biogenesis during ER stress [55,56].

When the UPR fails to restore cellular homeostasis, the cell prepares for apoptosis by activating CHOP, NF-kB, BiP, and XBP1 via the ATF6 signaling pathway [57]. CHOP activation is associated with the misfolded and unfolded proteins during ER stress, oxidative stress, and cell death under amplified insulin demand, as in type II diabetes [6]. In addition, Chen et al. in 2020 [58] reported that ATF6 activation via ER stress-induced cell death and inflammation can be alleviated by treatment with miR-149. 

When ER stress occurs in a cell that results in sorcin (a Ca^2+^-binding protein that helps to maintain Ca^2+^ homeostasis in ER) dysfunction, it also increases the ATF6 activity, which results in the advancement of insulin dysfunction, obesity, and type II diabetes [59]. Obesity and type II diabetes are associated with high-fat and high-glucose concentrations, which lead to beta-cell dysfunction in the pancreas via ATF6 [60]. Compared to the other two arm sensors (IRE1 and PERK) of ER stress, the ATF6 response in diabetes remains controversial [61] due to the lack of receptors on ATF6.

Although various signaling pathways for diseases, such as diabetes, have been identified since the 1970s to date, as listed in Table 1, the exact mechanisms involved in type II diabetes remain to be fully elucidated, particularly the structures of the three arm sensors, the complex downstream signaling interactions and intersections, and the reorganization of the noncanonical ER membrane. Many disorders, such as diabetes, cancer, neurodegeneration, autoimmunity, and obesity, are associated with elevated ER stress levels. The various cascade activations of the ER stress response likely depend on particular pathophysiological processes and disorders. Therefore, molecules that can stimulate or inhibit specific ER stress response pathways need to be studied to achieve better therapeutic outcomes in humans.

**Figure 2:**
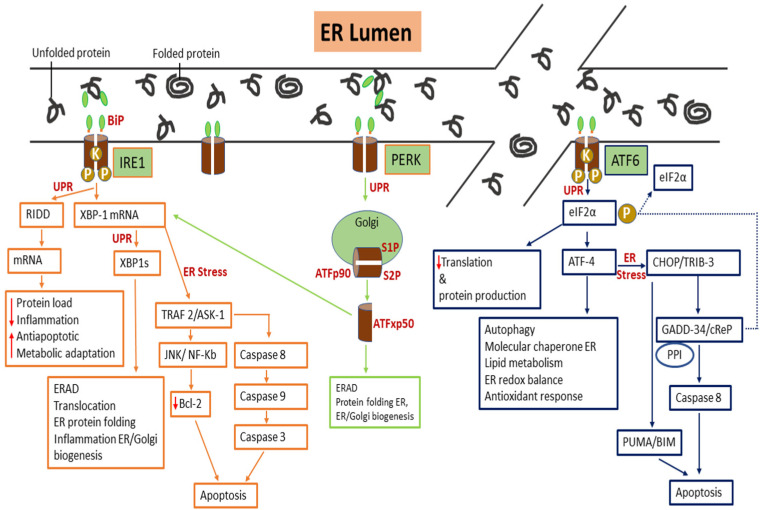
The three canonical arm sensors (IRE1, PERK, and ATF6) and BiP. The canonical arms are activated when unfolded proteins accumulate inside the ER lumen, leading to BiP dissociation. The downstream signaling activates IRE1 by performing intron splicing of XBP1 mRNA to produce active spliced XBP1s, which bind to the promoter regions of numerous genes involved in manufacturing chaperones and ERAD proteins to restore normal protein homeostasis. The phosphorylation of PERK leads to the activation of eIF2α, resulting in the reduction of protein translation, favoring an increase in the expression of ATF4. ATF-6p90 moves to the Golgi body, where it undergoes proteolytic cleavage. ATP-6p50 moves to the nucleus and activates chaperones, which help in the protein folding process. Abbreviations: Inositol-requiring kinase-1 (IRE1), Activating transcription factor 6 (ATF6), Double-stranded RNA protein kinase-like ER kinase (PERK), X-box-binding protein 1 (XBP1), X-box-binding protein 1 splicing (XBP1s), Binding immunoglobulin protein (BiP), Endoplasmic reticulum-associated degradation (ERAD), Regulated Ire 1-dependent decay (RIDD), TNF-associated factor 2 (TRAF2), Apoptosis signaling kinase 1 (Ask1), Site one protease (S1P) and Site two protease (S2P), c-Jun N-terminal kinase (JNK), Nuclear factor-kappa B (NF-κB), Activating transcription factor 4 (ATF4), C homologous protein/enhancer-binding protein homologous protein (CHOP), eukaryotic initiation factor 2 alpha (eIF2α), Tribbles-like protein 3 (TRB3), Protein phosphatase 1 (PP1), Constitutive repressor of eIF2α phosphorylation (CReP), B-cell lymphoma protein-2 of family proteins (Bcl-2),Growth arrest and DNA damage-inducible protein (GADD34), Bcl-2 interacting mediator of cell death (BIM), and p53 upregulated modulator of apoptosis (PUMA).

The three arm sensors (IRE1, PERK, and ATF6) are activated when BiP dissociates itself from them in the presence of misfolded and unfolded proteins in the ER lumen. All of them initiate downstream signaling via transcription factor generation and other associated factors to resolve the misfolded and unfolded protein load in the lumen. The UPR seeks to restore ER protein folding homeostasis and promote cell survival by modifying the production and translational demand of the transcription factors. If the UPR cannot overcome ER stress, mechanisms that encourage cell death are activated. These mechanisms are denoted in Figure 2.

## 4. ER Stress Response to Insulin Dysfunction

Cell survival, growth, proliferation, differentiation, lipid metabolism, glucose metabolism, and vascular functions require insulin action. Insulin exerts its function by communicating with the alpha and beta subunits of its receptor, leading to tyrosine autophosphorylation, which initiates its cascade of signals [4]. This signaling is mediated via phosphatidylinositol-3-kinase (PI-3K/Akt), with Ras/MAPK regulating glucose uptake, pro-survival, and cell differentiation and growth [69].

Insulin dysfunction and increased blood glucose levels are associated with obesity and type II diabetes [4]. Obesity can induce ER stress due to the phosphorylation of c-Jun N-terminal kinase (JNK) via IRE1α. This facilitates the alteration of an insulin signaling cascade with the resultant effect of insulin dysfunction and, ultimately, type II diabetes [9]. ER stress is a central molecular element linked to insulin dysfunction in obesity and diabetes [4,66]. ER stress indicators were found to be high with a high-fat diet and obese mice [70]. Furthermore, ER stress can lead to insulin dysfunction by compromising insulin signaling transduction and inhibiting Akt phosphorylation [70]. 

Panzhinskiy et al. in 2013 [71] demonstrated that ER stress led to insulin dysfunction by inhibiting insulin-stimulated sugar absorption. Regarding the molecular mechanisms of ER stress and insulin dysfunction, the activation of JNK and the expression of tribble-like protein 3 via IRE1 and PERK have been gaining attention in type II diabetes and obesity [9]. Villalobos-labra et al. in 2018 [9], reported that obesity was accompanied by insulin dysfunction due to ER stress activation in obese pregnant women. This observation was linked to fetoplacental endothelial dysfunction. In addition, the nitric oxide (NO) impairment observed in obese children was attributed to insulin dysfunction, which is a hallmark of endothelial dysfunction. 

Endothelial dysfunction in HUVECs can be resolved by administering molecular chaperone inhibitors of ER stress [72]. Obesity-induced endothelial dysfunction, which is closely linked with insulin dysfunction, is attributed to ER stress [66]. The ER plays a vital role in normal insulin functioning; however, ER stress modifies insulin sensitivity negatively, which causes insulin dysfunction [67,73]. Thus, a detailed understanding of ER stress and insulin dysfunction’s etiology can help find a novel treatment for type II diabetes. In addition, the direct and indirect effects of ER stress activation in relation to insulin dysfunction need to be comprehensively understood.

## 5. ER Stress Response to Oxidative Stress

Oxidative stress is a complex process that involves the excessive synthesis and availability of ROS, which are beyond the scavenging ability of the antioxidants in a cell. Free radicals in cells exist in the form of reactive nitrogen species and ROS; many are metabolic byproducts of a cell [74]. In addition to their physiological function in appropriate amounts, ROS also play a vital role in the etiology of many ailments, such as diabetes and obesity. However, their overproduction can hinder the physiological mechanisms in many disorders related to oxidative stress, which leads to apoptosis [74].

ER stress is directly related to oxidative stress and contributes to endothelial dysfunction [75]. The preservation of ER homeostasis is closely linked to a cell’s oxidative status. The major ROS produced by the ER is the H_2_O_2_ produced during protein folding in the ER lumen [76]. However, nicotinamide adenine dinucleotide phosphate oxidase 4 (NOX4) is also a well-known resident of ER, which produces H_2_O_2_ and superoxide anions [77]. The ER lumen is excessively oxidized due to the reduced amount of glutathione (GSH) relative to the cytosolic chamber to enable the creation of native disulfide bonds [78]. 

Under physiological conditions, GSH accounts for approximately 98% of the total GSH. Oxidized glutathione (GSSG) can be catalyzed back to GSH by an enzyme called glutathione reductase [79]. Hence, GSH is regenerated for cellular antioxidant defense, especially during the protein folding process [66]. In addition to GSH, other essential antioxidants that offer protection against oxidative insults to ER include glutathione peroxidase, peroxiredoxin 4, and ascorbate peroxidase [80].

In diabetes, there is an intensive demand for protein production, which leads to the formation of an extreme non-native disulfide bridge. This is due to the excessive consumption of GSH, which is responsible for protecting the cells from ROS. The ER–ROS association is regulated via signaling pathways involving Ca^2+^, ER oxidoreductase 1 alpha (ERO-1α), NOX4, cytochrome P450 reductase, and protein disulfide isomerase (PDI) [81]. The loss of GSH reserves within a cell results in amplified oxidative stress [75]. Moreover, ROS (due to hyperglycemia-induced oxidative stress) inhibit the disulfide isomerase enzyme, which results in the formation of misfolded and unfolded proteins [82]. 

These proteins cause the degradation of ATP. This results in higher glucose utilization to encourage mitochondrial oxidative phosphorylation, which raises ATP production, leading to enhanced ROS. However, the aggregation of misfolded and unfolded proteins in the ER lumen results in Ca^2+^ leakage into the cytosol, causing an increase in its concentration and ROS production from the mitochondria. The physical contact between mitochondria and the ER has been reported to have IRE1 or PERK in MAM [83]. 

This association can increase ROS generation via PERK during ER stress [84]. The leakage of considerable Ca^2+^ via MAM results in the blocking of complex III in mitochondria, with the resultant effects of electron leakage and amplified ROS production [85]. The depletion of Ca^2+^ from the ER lumen can induce more ER stress and oxidative stress in a vicious cycle [66].

Wu et al. in 2019 [86], showed the effect of homocysteine in triggering endothelial dysfunction by attenuating the ER redox balance. Homocysteine exhibits several effects due to its sulfhydryl group, which is very reactive. It can enhance ROS generation via NADPH oxidase in endothelial cells as well as the uncoupling of endothelial nitric oxide synthase (eNOS) [87,88,89]. High levels of ROS can damage the DNA and protein, thus, contributing to inflammation and apoptosis [90]. In addition, an increased accumulation of homocysteine can generate ER stress and trigger an ER stress response. Moreover, growing evidence suggests that oxidative protein folding in the ER forms the basis for overoxidation of ER and stress, which is referred to as ER oxidative stress [86,91,92]. 

Therefore, to avoid excess ROS build-up and ER oxidative stress, ERO-1α activity should be strictly controlled. Homocysteine can trigger mRNA transcription for ERO-1α through increased interactions with hypoxia-inducible factor-1 alpha in connection with the ERO-1α promoter region. ERO-1α is activated by homocysteine allosterically, which eventually leads to a decrease in PDI expression and the ER redox state, thus, decreasing the ratio of GSH/GSSG. Overall, the amplified activity of ERO-1α results in ER stress, the overproduction of H_2_O_2_, and inflammation [86].

The endothelium is unstable in type II diabetes, leading to immune cell dysregulation [24,93]. ROS are produced by these immune cells via a respiratory outburst that changes the endothelium’s integrity [94]. These variables generate oxidative stress and ROS in the endothelial cell, which encourages inflammatory conditions and endothelium dysfunction. Moreover, an excessive increase in ROS level leads to amplified levels of interleukin-1, IL-6, TNF α, and the expression of cellular adhesion molecules, such as vascular cell adhesion molecule-1, which contributes to complications related to type II diabetes [24,95]. In addition, inflammatory actions cause insulin dysfunction, diabetes progression, and amplified oxidative stress [96]. 

**Figure 3:**
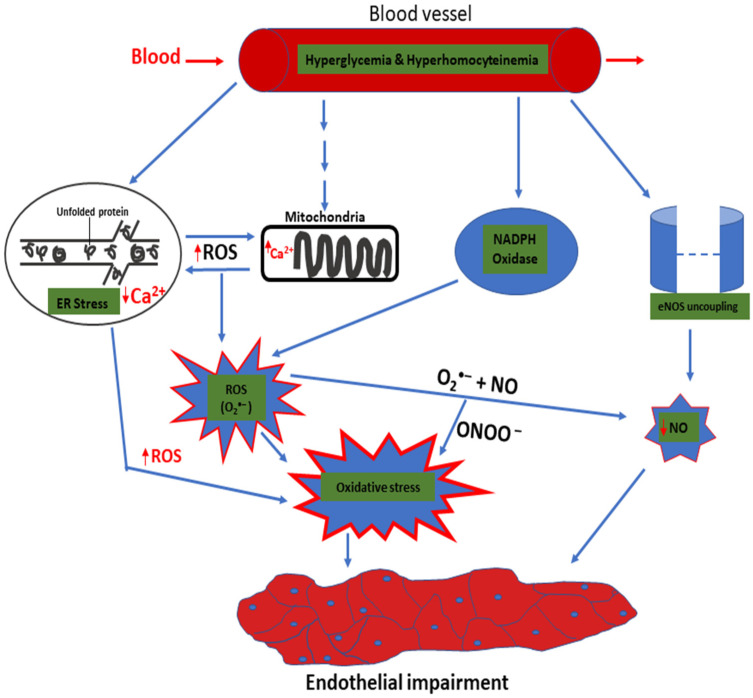
Hyperglycemia- and hyperhomocysteinemia-induced oxidative stress mechanisms lead to endothelial dysfunction. Hyperglycemia and hyperhomocysteinemia cause endothelial dysfunction, which contributes to vascular pathology. In addition, the presence of high-level glucose and hyperhomocysteinemia in the blood can cause the ER to sense it as metabolic distress, leading to the increased accumulation of unfolded protein in the ER lumen. The chronic accumulation of unfolded protein triggers ER stress. Furthermore, ROS production from the ER induces mitochondria and NAD(P)H oxidase to generate more ROS. The overall effect of this is that it causes eNOS uncoupling, leading to a reduction in NO production. The decrease in NO bioavailability ultimately leads to endothelial impairment. Abbreviations: Reactive oxygen species (ROS), Endoplasmic reticulum (ER), Nicotinamide adenine dinucleotide phosphate (NADPH), Endothelial nitric oxide synthase (eNOS), Calcium (Ca^2+^), Nitric oxide (NO), and Superoxide anion (O_2_^−^).

ROS destroy proteins, lipids, and DNA and activate cellular transcriptional alterations, which facilitate insulin dysfunction. Thus, insulin dysfunction is initiated, resulting in chronic hyperglycemia with the consequences of developing type II diabetes. Several pathways play a vital role in the generation of ROS, including ER stress. ER stress and the mitochondria also produce a high amount of ROS in a vicious cycle. The increased amount of ROS produced leads to a decrease in the bioavailability of NO and amplified oxidative stress, resulting in endothelial dysfunction. The peroxynitrite formed also contributes to the development of endothelial dysfunction. These are highlighted in Figure 3.

## 6. Therapeutic Potential of Targeting ER Stress

In recent years, considerable efforts have been made to create novel molecules to avert ER stress and enhance ER homeostasis. These molecules have been reported to help improve the glucose metabolism, endothelial dysfunction, and insulin function in diabetes [97]. Several studies have demonstrated the association between diabetes and ER stress [70,98,99,100,101]. Glembotski, in 2014 [102] indicated that the stimulation of UPR and ER stress is implicated in the pathogenesis of many cardiovascular diseases, such as heart failure, atherosclerosis, obesity, and diabetes. 

Thus, pharmacological interventions targeting the UPR pathways and ER stress, such as tauroursodeoxycholic acid (TUDCA) and sodium phenylbutyrate (SPB) belonging to chaperone molecules, can be harnessed to treat such conditions. These chaperones enhance the ability of ER protein folding to control ER stress and interfere with the downstream signaling pathways of the three arm sensors (i.e., PERK, IRE1, and ATP-6). Previous research reported that PERK operates as a metabolic sensor in cells and controls protein folding and secretion. 

PERK-knockout mice also exhibit decreased insulin content [103]. However, the enhanced expressions of BiP, phosphorylated PERK, and eIF2α are associated with diabetes. Logue et al. in 2018 [104] and Zhao et al. in 2018 [105] showed that the IRE1–XBP1 cascade controls cancer progression during pancreatic, prostate, and breast tumors. Pharmacological molecules affecting the IRE1 pathways may have therapeutic potential. This is because these pathways activate the inflammatory signals by stimulating NF-κB in many tissues through metabolic impairments, as in the cases of diabetes and obesity [106,107].

The Food and Drug Administration has approved the use of molecular chaperones, SPB, and TUDCA to improve ER homeostasis and decrease ER stress [107]. They also approved the use of certain antihypertensive drugs, such as Guanabenz, to reduce ER stress [66]. Furthermore, some molecules can activate GRP 78 in animal models. Zhang et al. in 2011 [108] indicated the use of valproic acid for treating seizure-protected epithelial cells from cell death in an ischemic model. 

Valproic acid also increases GRP 78 expression, decreases CHOP expression, and stimulates caspase enzymes. Tsaytler et al. in 2011 [109] reported that a centrally acting α2-adrenergic receptor agonist called Guanabenz prolonged eIF2α stimulation by inhibiting its dephosphorylation through phosphatase GADD34-selective inhibition. Another study reported that the angiotensin receptor blocker telmisartan can avert cell death via the IRE1α axis [110]. Xu et al. in 2012 [111] reported that verapamil, a calcium channel blocker used in islet cells, and murine models of type II diabetes can inhibit the genes for proapoptosis. Calcium channel blockers can be further explored to validate whether they can hinder the three arm sensors of ER stress.

## 7. Conclusions

Emerging evidence supports the roles of UPR and ER stress in the pathogenesis of pancreatic beta-cell dysfunction, insulin resistance, and apoptosis in type II diabetes. It is now believed that ER stress-related diseases, including type II diabetes, result from the apoptosis of stressed cells owing to the interplay of the complex of three arm sensors (IRE1, PERK, and ATF6). Therefore, understanding the UPR, ER stress, and their roles in the pathophysiology of diabetes can help to establish novel therapeutic strategies for the prevention and management of ER stress and ultimately diabetes.

## Figures and Tables

**Table 1:** Summary of studies on the evolution and advancement of the endoplasmic reticulum.

Year	Reference	Evolution and Advancement	Findings
1897	[62]	Ergastoplasm	First observed with a light microscope and named Ergastoplasm
1945	[16]	Lace-like reticulum	Lace-like reticulum extended into the thin margin and even into the fine processes of the cell from the denser center.
1953	[63]	Reticulum, which means “network”	Reticulum (network) was applied to describe this fabric of membranes
1954	[17]	Endoplasmic reticulum (RER and SER)	ER as a network of cavities enlarges into relatively vast and flattened vesicles described as cisternae.
1977	[35]	GRPs	Glucose-regulated proteins (GRP-78 and GRP-95) because the amount of the proteins is influenced by the presence or absence of glucose. These proteins play an important role in regulating glucose utilization in cultured cells.
1983	[36]	BiP	At least some H-chains are bound to a protein other than L-chain. Here, we show that the protein (which we term immunoglobulin heavy-chain binding protein, BiP) binds noncovalently to free IgH, but not to IgH associated with IgL.
1986	[37]	GRP and BiP were found to be the same	Identical with two previously described proteins: GRP78, whose synthesis is induced by glucose starvation, and BiP, which is bound to immunoglobulin heavy chains in pre-B cells.
1989	[39]	GRP and BiP were associated with misfolded and unfolded proteins	Increased factor VIII synthesis was correlated with an 80-fold increase in GRP78 mRNA and a 10-fold increase in GRP94 mRNA.
1993	[40]	IRE1	IRE1 is essential for cell viability under stress conditions that cause unfolded proteins to accumulate in ER. IRE1 encodes a transmembrane serine/threonine kinase that we propose transmits the unfolded protein signal. IRE1 is also required for inositol prototrophy.
1998, 1999	[44,45]	PERK and ATF6	PERK, a gene encoding type I transmembrane ER-resident protein, has a lumenal domain similar to the ER-stress-sensing lumenal domain of the ER-resident kinase IRE1 and a cytoplasmic portion that contains a protein-kinase domain most similar to that of the known eIF2α kinases, PKR and HRI. In response to various environmental stresses, eukaryotic cells downregulate the protein synthesis through phosphorylation of the alpha subunit of eukaryotic translation initiation factor 2 (eIF2α). In mammals, the phosphorylation is carried out by eIF2α kinases PKR and HRI.
2001	[42]	XBP1	The transcription factor XBP1, a target of ATF6, as a mammalian substrate of such an unconventional mRNA splicing system showed that only the spliced form of XBP1 can activate UPR efficiently.
2006	[21]	Organized ER into a membrane structure	ER has distinct morphological domains composed of sheets and tubules, which differ in their characteristic membrane curvature.
2008	[64]	ER stress	Paraoxonase-2 (PON2) is a ubiquitously expressed antioxidative protein primarily found in ER. PON2 overexpression provides significant resistance to ER-stress-induced caspase-3 activations.
2012	[6,65]	ER stress linked to diabetes	Both chronic hyperglycemia and hyperlipidemia, known as critical causative factors of type II diabetes, disrupt ER homeostasis to induce unresolvable UPR activation and β-cell death. ER stress can be an essential contributor to diabetes-related vascular complications.
2019	[24,66]	ER stress linked to diabetes	The implication of mitochondria in insulin release and the exposure of pancreatic β-cells to hyperglycemia make them especially susceptible to oxidative stress and mitochondrial dysfunction. ER stress response is now recognized as a converging molecular link that connects insulin resistance, lipid metabolism disturbances, cell death, and oxidative stress to endothelial dysfunction.
2020, 2021	[67,68]	ER stress linked to diabetes	Chronic hyperglycemia, hyperinsulinemia, increased proinflammatory cytokines, and free fatty acids found in diabesity can lead to ER stress. The inflammatory response to the damage induced by hyperglycemia and ROS becomes chronic as diabetes progresses and constitutes the leading cause of vascular complications.

## Data Availability

Not applicable.
